# The Cause of Rickets

**Published:** 1904-03-26

**Authors:** 


### THE CAUSE OF RICKETS.

Dr. Nathan[1] gives a summary of the chief theories which have been held to account for the occurrence of rickety manifestations in children, together with the experimental data upon which such explanations have been based or by which they have been refuted. The most constant and characteristic lesions of rachitis are, of course, those affecting the skeleton, and consist essentially of defective calcification of the cartilage and newly formed bone tissue. That the cause which leads to this failure of deposition of calcium salts is a general constitutional condition is indicated not only by the fact that the skeleton is uniformly affected, but also by the presence of concomitant symptoms outside the skeletal structures, *e.g.* bronchitis, intestinal disturbance, and enlarged spleen; unless, indeed, it be assumed that absence of sufficient calcium from the blood is the primary cause of all the symptoms.

Thus the subject can be studied from two standpoints; it can be assumed that rickets is due to a constitutional anomaly, one of the symptoms of which is an inhibition of normal calcium deposition in the growing bones, or it may be supposed that a deficiency in the amount of calcium within the organism is instrumental in producing not only the faulty calcification but also the other lesions.

With regard to the second alternative, it has been demonstrated experimentally that total deprivation of calcium from the food will cause typical rachitic bone lesions in growing animals. Though of scientific interest, this experiment does not materially advance our knowledge of the normal etiology, since children will develop marked rickets when fed on artificial foods in which there is excess of calcium salts, and will recover from the affection on an altered diet containing a smaller quantity of these salts than were present in the food which was primarily responsible for the illness. Hence it has been argued that it is not the ingestion but the absorption of calcium that is deficient, and various theories have been propounded to account for this supposed defect, notably, the one which attributes the faulty process primarily to deficient HCl secretion in the stomach. Dr. Nathan states, however, that there is abundant evidence to show that the HCl secretion is not absent or deficient in rickets, and hence this theory is untenable. He holds in view of these facts that the theories of deficient calcium ingestion and calcium absorption must be discarded, and that the basis for future research must be the assumption of a defect of metabolism, which, besides causing other abnormalities, is also responsible for the improper calcification in the growing bones.

[1] Med. News, Feb. 27.

